# A Case Report of Peritoneal Tuberculosis: A Challenging Diagnosis

**DOI:** 10.1155/2018/4970836

**Published:** 2018-01-11

**Authors:** Dilara Bulut Gökten, Bilal Katipoglu, Ekrem Basara, Ihsan Ates, Nisbet Yılmaz

**Affiliations:** Department of Internal Medicine, Ankara Numune Training and Research Hospital, Ankara, Turkey

## Abstract

Peritoneal tuberculosis is a disease which can mimick malignancy especially in women who present with ascites and elevated CA125 levels. It should always be considered in differential diagnosis, but the diagnosis is rarely easy for clinicians. A young female patient who presented with abdomen tenderness and diagnosed with peritoneal tuberculosis as a result of performed tests is discussed hereby in the case report. We expect that this case report adds to the existing literature on this subject.

## 1. Introduction

Tuberculosis which is one of the ancient diseases is known to affect human health and is caused by the bacteria *Mycobacterium tuberculosis*. This old disease may be fatal within just 5 years in more than 50% of cases [1]. Extrapulmonary tuberculosis (ETB) accounts for 18.7% of all tuberculosis patients in the USA, and peritoneal tuberculosis is not a common form of it. It is seen just in 4.7% of all patients [2]. Turkey is a country that has a moderate level of TB. With the implementation of the Health Transformation Programme, our health indicators reached the same level as those in upper-income countries. Tuberculosis prevalence decreased from 38 per 100,000 population in 2002 to 24 per 100,000 population in 2011 in provinces and district centres [3]. According to the World Health Organization (WHO) Global Tuberculosis Report 2017, estimated tuberculosis incidence is 6 (5.1–6.9) per 100,000 in the female group, 8 (6.8–9.2) per 100,000 in the male group, and 14 (12–16) per 100,000 population totally in Turkey [4]. Also, peritoneal tuberculosis is a disease which can mimick malignancy especially in women who present with ascites and elevated CA125 levels. Ascites of tuberculosis is an exudative form just as in malignancy cases. Moreover, they share many similarities in symptoms and radiology and laboratory results. True diagnosis with and correct follow-up can decrease patient morbidity and deaths. Peritoneal tuberculosis is a rare entity in the literature and will be discussed in this case report.

## 2. Case Report

**Figure 1:**
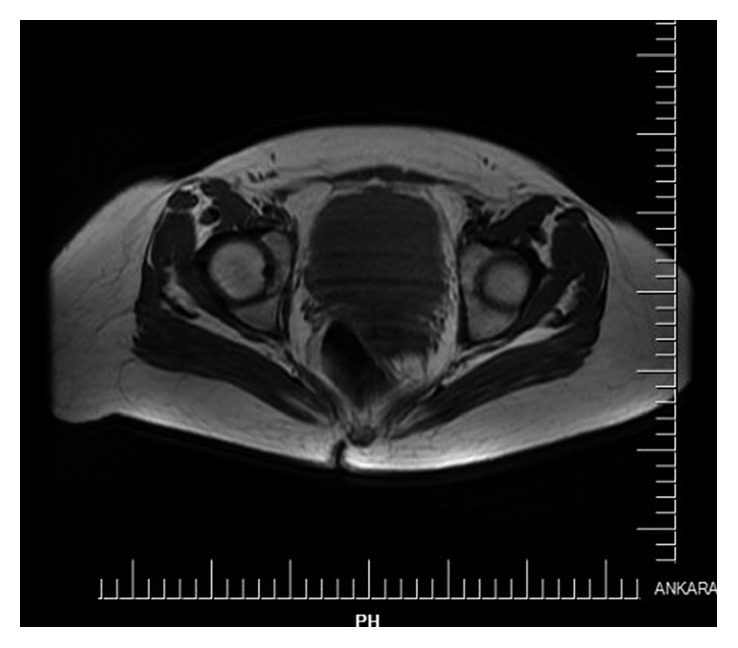


A 16-year-old female was admitted to the hospital with a 7-day history of tenderness in the abdomen who was referred from another centre to us with reported ascites in ultrasonography. No chronic diseases, no alcohol use, no family history, no herbal agents, or no suspected drug use were reported. Vital signs of the patients were in the normal ranges with 36.3 body temperature, 120/70 arterial blood pressure, and 82/min heart rate. Our physical examination referred ascites in the abdominal region, and there was no other abnormal finding. Our laboratory results reported no anemia, no white blood cell elevation, and no thrombocyte abnormality. Her peripheral smear was normal with no atypical cells. No renal function or hepatic function abnormality and no electrolyte abnormality were also reported. CRP was 39 (normal range: 0–5 mg/L) and erythrocyte sedimentation level was 42 (normal range: 0–20 mm/h). Her chest X-ray was completely normal with no infiltrations or effusions. Her abdominal ultrasonography reported abdominal free liquid deposition, septations in the fluid accumulation, and multiple implants in peritoneal surfaces in hepatic diaphragmatic region and right paracolic area, in which the biggest one was 16 millimetres. Endometrium thickness was in the normal range, but in the left ovary, there was a septated cyst with dimensions of 39∗27 millimetres (hemorrhagic cyst?). We suspected from ovarian pathology and performed paracentesis and sent this fluid to pathology laboratory. Pathology reported inflammatory cells which are rich in lymphocytes. There were no malignant cells in the fluid. According to the laboratory results, serum albumin ascites gradient was <1.1, and this showed us that the fluid was exudate. The patient's thyroid function tests were completely normal. Laboratory investigations showed the following values: AFP level 2.48 (normal range: 0–7 ng/mL), CEA level 0.39 (normal range: 0–3.8 ng/mL), CA15-3 level 26.15 (normal range: 0–26.4 U/mL), CA19-9 level 1.14 (normal range: 0–27 U/mL), and elevated CA125 level of 107.5 (normal range: 0–35 U/mL). Brucella markers and viral markers were negative. Although the patient had no encounter with any tuberculosis patient, we performed tuberculin skin test for differential diagnosis. As a result, it was anergic. Tuberculosis quantiferon PCR was negative, and ascites fluid direct microscopic examination revealed that there were no tuberculosis bacilli and acid-fast stain was negative. Tomography of the thoracoabdominal region revealed nasopharyngeal soft tissue with the dimensions of 20∗18 millimetres, bilateral lymphadenopathies in the levels of 1a, 1b, 2, 3, 4, and 5, and right supraclavicular area in the cervical region. The nasopharyngeal biopsy revealed just lymphoid hyperplasia. Paratracheal and subcarinal lymphadenopathies were also seen, and the biggest one was 20∗13 millimetres. Also, there were bilateral anterior diaphragmatic and right cardiophrenic lymphadenopathies. Abdomen tomography showed fluid accumulation and hepatic peritoneal surface nodular lesions. There were a reticular density increase in the omental area and a cyst in the left ovary with the dimensions of 23∗18 millimetres. There were also lymph nodes in the mesenteric fatty tissue. Although malignancy was important for differential diagnosis, ADA (adenosine deaminase) level was 62 (normal range: 0–30 U/L). These results strongly supported tuberculosis. Then, we sent peritoneal fluid to the pathology laboratory again and no malignant cells were reported. Abdomen magnetic resonance imaging reported bilateral cysts in both the ovarian regions, bilateral expanded fallopian tubes, nodular opacities in the peritoneal region, and ascites (Figure 1). We performed trucut biopsy from nodular opacities in that peritoneal surface, and pathology laboratory reported granulomas with histiocytic cells in that specimen. We communicated with infectious diseases committee and started four-agent tuberculosis treatment (isoniazid, ethambutol, pyrazinamide, and rifampin) to the patient. After 6 months of treatment, she had good clinical response and ascites were completely absent.

## 3. Discussion

Peritoneal tuberculosis is a very rare disease in developed countries but always should be considered in developing countries. It accounts for 0.1% to 0.7% of tuberculosis cases [2]. It manifests the symptoms such as fever, loss of weight, infertility, abdominal and pelvic pain, and irregularities of menstruation. The other symptoms include ascites, adnexal masses, and increased level of CA125.

This tuberculosis case is very difficult to distinguish from abdominal malignancy cases. Symptoms can be similar, for example, weight loss, fever, abdominal pain, and abdominal swelling. CA125 levels can be high in both these cases. Moreover, radiological imaging of abdomen is very similar in both these cases such as ascites, nodular irregularities in the peritoneal surface, adnexal and fallopian masses, and septated and multiloculated ovarian cysts [5]. Also, there are some clues to distinguish these cases, peritoneal carcinomatosis and ovarian cancer are usually seen in older ages according to tuberculosis cases. CA125 levels are usually higher in peritoneal carcinomatosis cases. But, it should be noted that CA125 can elevate to about 10-fold of normal value in patients who have peritoneal tuberculosis [6].

Our case was mimicking ovarian cancer completely. Thus, peritoneal tuberculosis must always be considered in differential diagnosis of ovarian carcinoma especially in the developing countries or underdeveloped countries.

In the literature, most of the cases have encounter with tuberculosis patients and have different symptoms, cavitations in the lung or pulmonary focus appearance. Additionally, the patients who have peritoneal tuberculosis also have other comorbidities like immunocompromisation, cirrhosis, renal failure, diabetes mellitus, and malignancy [7]. But, in our case, there were no symptoms other than ascites, no pulmonary complaints, no encounter with any tuberculosis patient, no pulmonary foci in the chest X-ray or physical examination, and also there were no other medical conditions which can be helpful to suspect tuberculosis.

There are a lot of diagnostic procedures for tuberculosis in the literature, but none of them is completely specific or sensitive. Radiologic imaging techniques are not sensitive or specific for diagnostic purposes. Ascitic fluid cytology has a low negative predictive value. Although the test for acid-fast bacilli in the peritoneal fluid is highly specific for the diagnosis, it lacks sensitivity. There are high false-negative rates for tuberculosis skin tests. New diagnostic procedures like PCR assay for bacteria could help to identify this subject, since they can decrease the time taken to get a true diagnosis and specially helpful when AFB test is negative. In our case, PCR, AFB, and cytology were performed from abdominal ascites fluid and were negative. We tried to identify *Mycobacterium tuberculosis* in the samples, but all of the sample materials had negative result (urine sample, peritoneal fluid, sputum, and blood). We also performed quantiferon test to the patient, and it was also negative. In the literature, it is said that the use of the quantiferon assay for the detection of TB infection in patients with active pulmonary tuberculosis yielded sensitivity of 86 %, specificity of 94 %, PPV of 16.7 %, and NPV of 96.1 % [8]. Quantiferon test is precious for latent tuberculosis infection.

Despite the fact that identification of mycobacteria in any material is the gold standard method to evaluate the disease, negative result of culture cannot exclude the tuberculosis diagnosis. Activity of ascitic fluid adenosine deaminase (ADA) has been proposed as a useful test for abdominal tuberculosis cases. In countries with a high incidence of tuberculosis, measurement of ADA may be a helpful screening test. A value of ADA higher than 0.40 uKat/l has 100% sensitivity and a specificity of 99% for diagnosing tuberculous peritonitis [9]. However, in developed populations with low incidence of tuberculosis and a high prevalence of cirrhosis, ascitic fluid ADA activity is good in accuracy but poor in sensitivity and imperfect in specificity. Fortunately, our case's ADA level is high enough to support a diagnosis of tuberculosis.

Like in our cases, in selected cases, performing tissue biopsy is appropriate to identify the disease. Since our case did not have any respiratory complaints that would make one consider tuberculosis, the tissue biopsies performed would eventually confirm the diagnosis. So, diagnosis of peritoneal tuberculosis is usually made by histology of biopsy material which reveals granuloma.

Extrapulmonary tuberculosis usually results from hematogenous or lymphogenous dissemination. Sometimes, infection directly extends from an adjacent organ. For instance, cutaneous tuberculosis can be acquired either exogenously (directly through injured skin) or endogenously (spreading from other organs). Skin trauma due to scratching easily introduces pathogens into the skin [10]. Peritoneal infection may represent seeding from abdominal lymph nodes or from salpingo-oophoritis. Tubercle bacilli may even spread to tendon sheaths (tuberculous tenosynovitis) by direct extension from adjacent lesions in bone or hematogenously from any infected organ.

Tuberculosis is also affected by current health situation of the human body [11]. Immunocompromised patients have to be evaluated on a case-by-case basis as patients with HIV infection are 26 to 31 times more likely to develop tuberculosis than patients without HIV infection. Also, diabetes is a common comorbidity in people with tuberculosis, and malnutrition increases the risk of tuberculosis. Tobacco smoking increases the risk of tuberculosis two- to threefold and is associated with poor TB treatment results.

About 20%–40% of patients with abdominal tuberculosis present with an acute abdomen and need surgical management. Surgery is essentially reserved for those with acute surgical complications including free perforation, confined perforation with abscess or fistula, massive bleeding, complete obstruction, or obstruction not responding to medical management. Fillion et al.'s study in a low prevalence country reported that, out of 86% presenting with abdominal symptoms, 76% underwent surgery, with 10% in an emergency setting [12]. Abdominal tuberculosis is generally responsive to medical treatment, and early diagnosis and management can prevent unnecessary surgical intervention.

## 4. Conclusion

By information given in this case report, one can understand that peritoneal tuberculosis can often mimick advanced ovarian cancer and peritoneal carcinomatosis. It should always be considered in differential diagnosis, but the diagnosis is rarely easy for clinicians. True diagnosis and then correct and careful follow-up can save the patient's life, and doctors should start the treatment as soon as possible.

## References

[1] Raviglione M. C., O’Brien R. J., Braunwald E., Fauci A. S., Kasper D. L. (2001). Tuberculosis. *Harrison Principles of Internal Medicine*.

[2] Peto H. M., Pratt R. H., Harrington T. A., LoBue P. A., Armstrong L. R. (2009). Epidemiology of extrapulmonary tuberculosis in the United States, 1993–2006. *Clinical Infectious Diseases*.

[3] WHO (2017). *Global Tuberculosis Report, WHO/HTM/TB/2017*.

[4] Ministry of Health (2011). *Turkey Combat against Tuberculosis 2011 Report*.

[5] O’Riordan D. K., Deery A., Dorman A., Epstein O. E. (1995). Increased CA 125 in a patient with tuberculous peritonitis: case report and review of published works. *Gut*.

[6] Choi C. H., Kim C. J., Lee Y. Y. (2010). Peritoneal tuberculosis: a retrospective review of 20 cases and comparison with primary peritoneal carcinoma. *International Journal of Gynecological Cancer*.

[7] Kaya M., Kaplan M. A., Isikdogan A. (2011). Differentiation of tuberculous peritonitis from peritonitis carcinomatosa without surgical intervention. *Saudi Journal of Gastroenterology*.

[8] Kobashi Y., Obase Y., Fukuda M., Yoshida K., Miyashita N., Oka M. (2006). Clinical reevaluation of the QuantiFERON TB-2G test as a diagnostic method for differentiating active tuberculosis from nontuberculous mycobacteriosis. *Clinical Infectious Diseases*.

[9] Hillebrand D. J., Runyon B. A., Yasmineh W. G., Rynders G. P. (1996). Ascitic fluid adenosine deaminase insensitivity in detecting tuberculous peritonitis in the United States. *Hepatology*.

[10] Saporito L., Florena A. M., Colomba C., Pampinella D., Di Carlo P. (2009). Prurigo nodularis due to *Mycobacterium tuberculosis*. *Journal of Medical Microbiology*.

[11] Bonura C., Di Carlo P., Spicola D. (2012). Rapidly growing mycobacteria in TB/HIV co-infection: a report of two cases focusing on difficulties in diagnosis and management. *New Microbiologica*.

[12] Fillion A., Ortega-Deballon P., Al-Samman S. (2016). Abdominal tuberculosis in a low prevalence country. *Médecine et Maladies Infectieuses*.

